# Ovarian serous adenocarcinoma identified during IVF: diagnostic approach, surgical management, and reproductive outcome

**DOI:** 10.1186/1477-7819-7-46

**Published:** 2009-05-14

**Authors:** David J Walsh, Eric Scott Sills, Lyuda V Shkrobot, Noreen C Gleeson, Mary N Sheppard, Anthony PH Walsh

**Affiliations:** 1 Division of Reproductive Endocrinology and Infertility, Department of Obstetrics and Gynaecology, School of Medicine, Royal College of Surgeons in Ireland, Dublin, Ireland; 2 The Sims Institute & Sims International Fertility Clinic, Dublin, Ireland; 3 Division of Gynaecologic Oncology, Department of Obstetrics & Gynaecology, Coombe Women's Hospital, Dublin, Ireland; 4 Department of Pathology, Royal Brompton Hospital, London, UK

## Abstract

**Background:**

To present a diagnostic evaluation and treatment strategy for serous adenocarcinoma of the ovary discovered during an in vitro fertilisation (IVF) sequence, and report on reproductive outcome after tumour resection and embryo transfer.

**Case presentation:**

Cycle monitoring in IVF identified an abnormal ovarian lesion which was subjected to ultrasound-guided needle aspiration. Cytology suggested malignancy, and unilateral oophorectomy was performed after formal staging. After surgery, the patient underwent an anonymous donor oocyte IVF cycle which established a viable twin intrauterine pregnancy. No recurrence of cancer has been detected in the >72 month follow-up interval; mother and twin daughters continue to do well.

**Conclusion:**

Suspicious adnexal structures noted during controlled ovarian hyperstimulation for IVF warrant assessment, and this report confirms the role of aspiration cytology in such cases. If uterine conservation is possible, successful livebirth can be achieved from IVF if donor oocyes are utilised, as described here.

## Background

Malignant ovarian neoplasms are uncommonly encountered during *in vitro *fertilisation (IVF). While response to gonadotropin treatment during fertility treatment is typically confined to assessment of follicular dimensions correlated with serum oestradiol levels, any abnormal ovarian morphology observed in this context should prompt careful evaluation and prompt referral to a gynaecologic oncologist. This is the first reported case in Europe of aspiration cytology used to identify ovarian serous adenocarcinoma during IVF, and highlights the role of this investigative approach for patients undergoing advanced reproductive treatments.

## Case presentation

**Figure 1:**
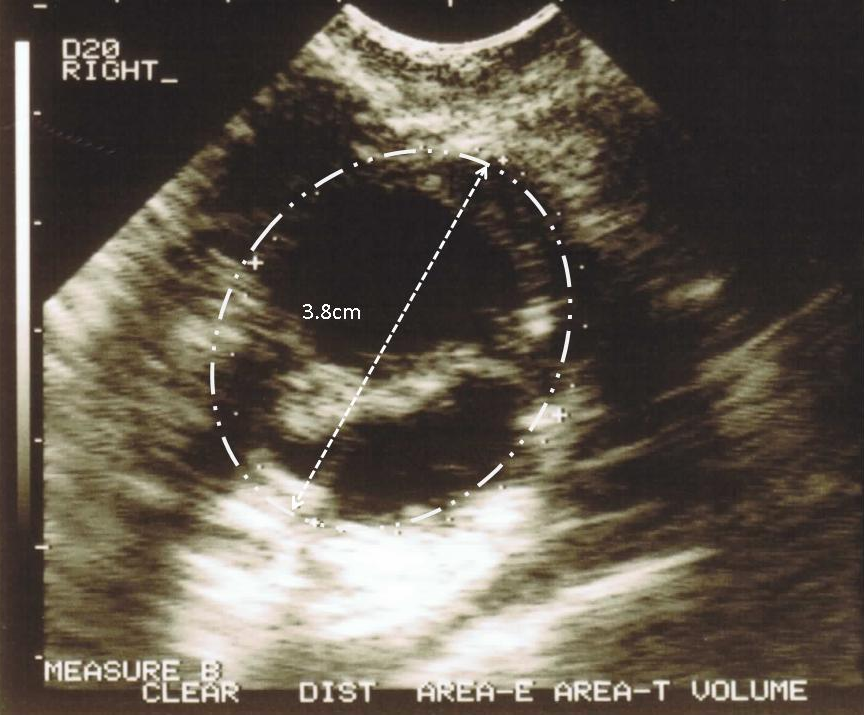
**Transvaginal ultrasound image of septated right ovarian cyst in IVF, which reappeared after puncture performed prior to gonadotropin therapy**. Aspirated fluid was consistent with borderline vs. well-differentiated ovarian serous adenocarcinoma.

A healthy 28 year-old nulligravida with polycystic ovary syndrome and no family history of breast or ovarian cancer was referred with her husband for reproductive endocrinology consultation. He was 31 and had a prior semen analysis suggesting asthenozoospermia (motility <40%). Hysteroscopy and laparoscopic ovarian drilling had been performed about six months before beginning fertility treatment. Bilateral tubal patency was confirmed and both ovaries appeared grossly unremarkable. Screening laboratory tests for both partners were normal and repeat semen analysis here found sperm concentration to be 100 M/ml, motility 60% and 35% abnormal forms (1992 WHO criteria). Based on these findings, the couple elected to undergo intrauterine insemination following ovulation induction with clomiphene citrate. After no pregnancy was achieved after three cycles, a simple 4 cm right ovarian cyst was noted and further ovulation induction was deferred until this lesion regressed. The cyst was essentially unchanged two months later, and serum CA-125 was 36.2 u/ml (reference range <35 u/ml), although the borderline elevation was thought to be secondary to recent exposure to fertility agents. By this time, the couple had elected to pursue IVF and, in anticipation of this, the cyst was decompressed by ultrasound-guided transvaginal needle drainage. While this cyst fluid was not specifically analysed, the ovaries now appeared grossly normal and an uneventful IVF cycle commenced. Seventeen oocytes were retrieved, and careful assessment of the right ovary identified a septated 3.8 cm cyst (Figure 1) which was aspirated separately from follicular fluid and collected oocytes. The structure was mapped to a similar location where the previous needle puncture and drainage had occurred. This time, the ovarian cyst fluid was submitted for formal cytologic evaluation. The patient had an uncomplicated day-three embryo transfer (*n *= 2), and there were three blastocysts available for subsequent cryopreservation.

**Figure 2:**
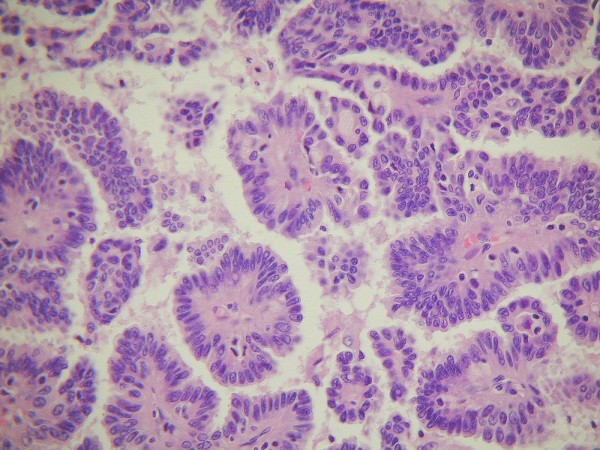
**Ovarian serous adenocarcinoma with finger-like papillae with fibrovascular core covered by multilayered cuboidal/columnar epithelium**. Haematoxylin and eosin, ×400.

The cytology data were returned five days after embryo transfer, and was consistent with borderline or well-differentiated serous adenocarcinoma. The patient was counselled and gynaecologic oncology referral was initiated. The pregnancy test from IVF was negative and 14 d after receiving the cytologist's report, the patient underwent laparotomy for unilateral right oophorectomy, left ovarian biopsy, omentectomy, appendectomy, and pelvic/para-aortic lymph node biopsy. Intraoperative pelvic washings were submitted for cytology and were negative. Staging showed benign tissue throughout, although a small focus of similar cancer was identified in the left ovary; at the patient's request pre-operatively this ovary remained *in situ*. The diagnosis of Stage IB ovarian serous adenocarcinoma of low malignant potential (Figure 2) was made, and the patient had an unremarkable post-surgical recovery.

The patient had monthly assessments by the gynaecologic oncologist who mandated frequent follow-up visits while ovulation induction was temporarily interrupted. This was coordinated with our IVF clinic, and numerous ultrasound studies were performed on her left ovary. The left ovary was removed by laparotomy 13 months after the right ovary and no additional abnormal cells were identified. Eight months later, the patient's frozen embryos were thawed and transferred but the pregnancy test was negative 14 d later. An anonymous donor oocyte IVF cycle commenced 16 months later and this resulted in a two-blastocyst transfer, following oncology clearance. A positive pregnancy test was noted 12 d after transfer and a viable twin intrauterine pregnancy was identified on transvaginal ultrasound on day 55. Her obstetrical course was uncomplicated until 31 weeks' gestation, when extreme oedema developed. Although she was normotensive and albuminuria was absent, moderately severe abdominal pain supervened and the patient was delivered by Caesarean at 34 1/2 weeks' gestation. During surgery, dense adherence of small bowel to the anterior uterine wall was noted and was regarded as the cause of the abdominal pain which resolved postoperatively. The patient remains cancer free for >72 months and her twin daughters (now age 4) continue to do well.

## Discussion

Frequent ovarian monitoring by transvaginal ultrasound is central to IVF patient evaluation, and this surveillance can occasionally result in the discovery of occult, subclinical cysts that would otherwise go undetected [1]. Even when complex ovarian cysts are incidentally noted at baseline ultrasound, the necessity of aspirating such lesions before IVF has been questioned. Indeed, an analysis of over 200 IVF patient cycles concluded that baseline cysts do not negatively affect reproductive outcome [2]. Endometriotic cysts and dermoids account for many of these cysts, and only two prior cases of ovarian cancer related to IVF – both from USA – appear in the literature [1,3].

Data on aspiration cytology of ovarian cysts developing in patients undergoing IVF treatment was considered rare a decade ago [3], and there has been little published on the topic since. The high false negative rate for nonfollicular lesions has limited the diagnostic value of aspiration cytology for many ovarian cysts [4] and information provided by ovarian cyst aspiration has been shown to correlate poorly with histology from tissue obtained at surgery [5]. Indeed, a four-year series comparing ovarian cyst cytology with histologic findings based on cases collected at a single centre reported 20% of cytology specimens as non-diagnostic [6]. Interestingly, aspiration cytology failed to determine the exact underlying nature of ovarian cysts in >50% of lesions when applied specifically to IVF patients, and an ovarian serous cystadenocarcinoma was the only malignancy identified [3]. Others have found aspiration cytology to be an accurate predictor of malignancy in cystic ovarian lesions, but have discouraged reliance on aspiration cytology results alone [7].

This case is only the third published report of ovarian cancer identified during IVF, and is the first to offer long-term follow up. However, several aspects of clinical management could have been different and warrant comment. First, cytologic examination of the initial ovarian cyst fluid would have suggested malignancy about a month earlier and would have justified abandonment of the planned IVF cycle. We subsequently modified institutional policy to mandate external cytology review for any ovarian cyst aspirates obtained here. Second, bilateral oophorectomy could have been performed during formal staging. This would have obviated the need for a second surgery for removal of the contralateral ovary, and arguably could have hastened the patients' enlistment into a donor oocyte programme for definitive fertility treatment. The possibility of bilateral oophorectomy was presented before the first laparotomy, and the patient was thoroughly counselled about potential malignant spread if this was not done. We also discussed the potential for malignant spread secondary to intraperitoneal spillage during cyst puncture. Even though a frozen embryo transfer remained a possibility, the patient did not wish to have both ovaries immediately removed. The tailored, multi-stage surgical approach described here was only possible with co-management by gynaecologic oncology and should not be undertaken without such support.

In summary, although aspiration cytology of ovarian cysts sometimes presents an unclear picture [8] it can help identify patients for whom oncology consultation is immediately indicated. We therefore support formal cytologic assessment of any suspicious complex ovarian lesion despite the recognised limitations of this approach.

## Consent

Written consent was obtained from the patient for publication of this case report. A copy of the consent is available with editor

## Competing interests

The authors declare that there are no competing interests.

## Authors' contributions

DJW was principal consultant for IVF, ESS was research consultant and reproductive endocrinologist, LVS was medical associate and chief ultrasonographer, NCG was gynaecologic oncologist and attending obstetrician, MNS was consultant pathologist, APHW conceived the research, prepared the manuscript and coordinated research & clinical teams. All authors read and approved the manuscript.
